# Supplementary material for: Boosting the stability of perovskites with exsolved nanoparticles by B-site supplement mechanism
Source: Nat Commun. 2022 Aug 8;13:4618. doi: 10.1038/s41467-022-32393-y (PMC9359987; doi:10.1038/s41467-022-32393-y)
Supplement: Supplementary file 1 — Supplementary Information [file 41467_2022_32393_MOESM1_ESM.pdf]

Boosting the stability of perovskites with exsolved nanoparticles by B-site supplement mechanism

Bo-Wen Zhang [1,5], Meng-Nan Zhu [1,5], Min-Rui Gao [1], Xiuan Xi [2], Nanqi Duan [1], Zhou Chen [3], Ren-Fei Feng [4], Hongbo Zeng [1], and Jing-Li Luo [1,2] *

Affiliation

1. Department of Chemical and Materials Engineering, University of Alberta, Edmonton, Alberta, T6G 1H9, Canada
2. College of Materials Science and Engineering, Shenzhen University, Shenzhen 518060, China
3. College of Materials, Xiamen University, Xiamen 361005, P.R. China
4. Canadian Light Source Inc., Saskatoon, Saskatchewan, S7N 0X4, Canada
5. These authors contributed equally: Bo-Wen Zhang, Meng-Nan Zhu.

* Corresponding author, Jing-Li Luo, E-mail: jingli.luo@ualberta.ca

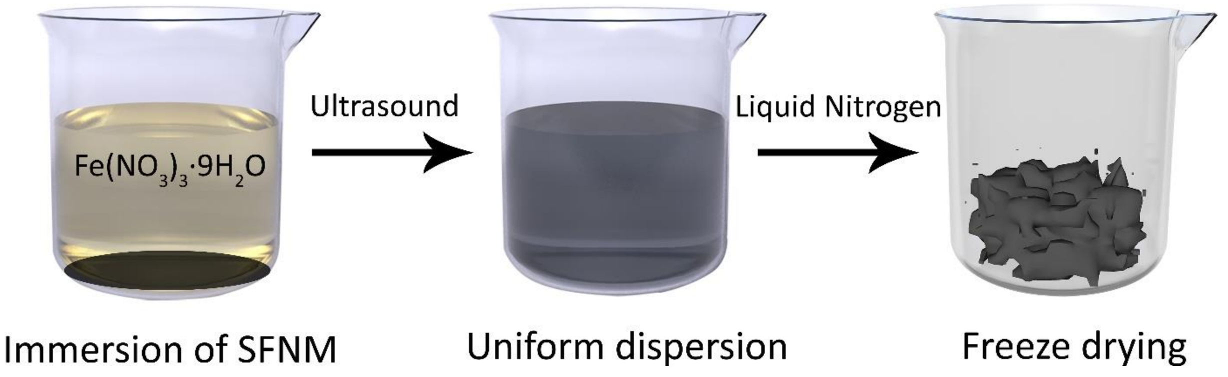

Supplementary Figure 1. Synthesis route of guest Fe deposition on the surface of SFNM for TIE-assisted exsolution process.

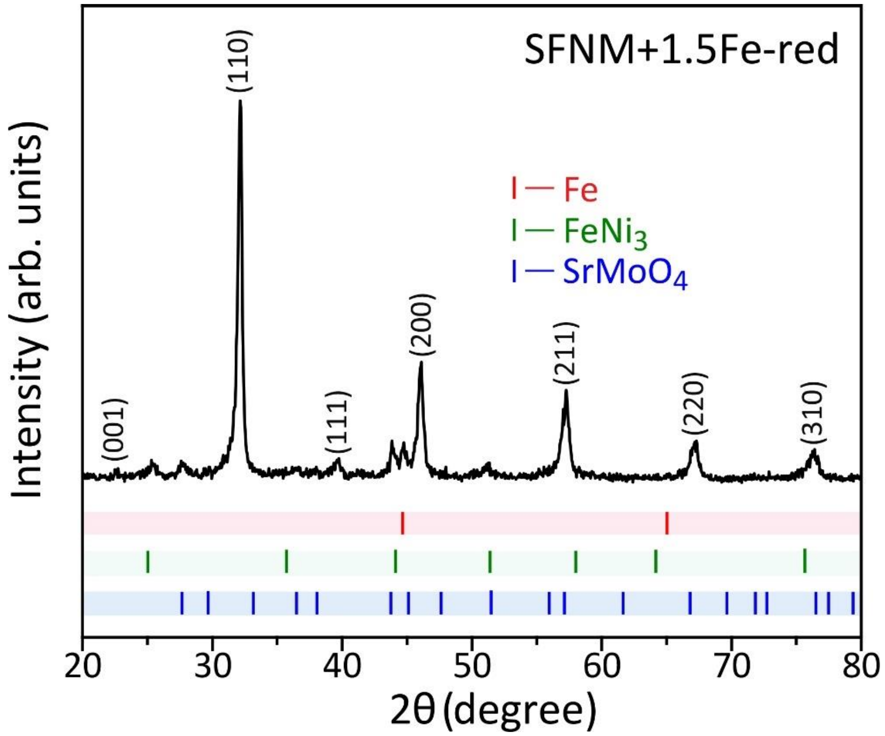

Supplementary Figure 2. XRD pattern of SFNM+1.5Fe-red.

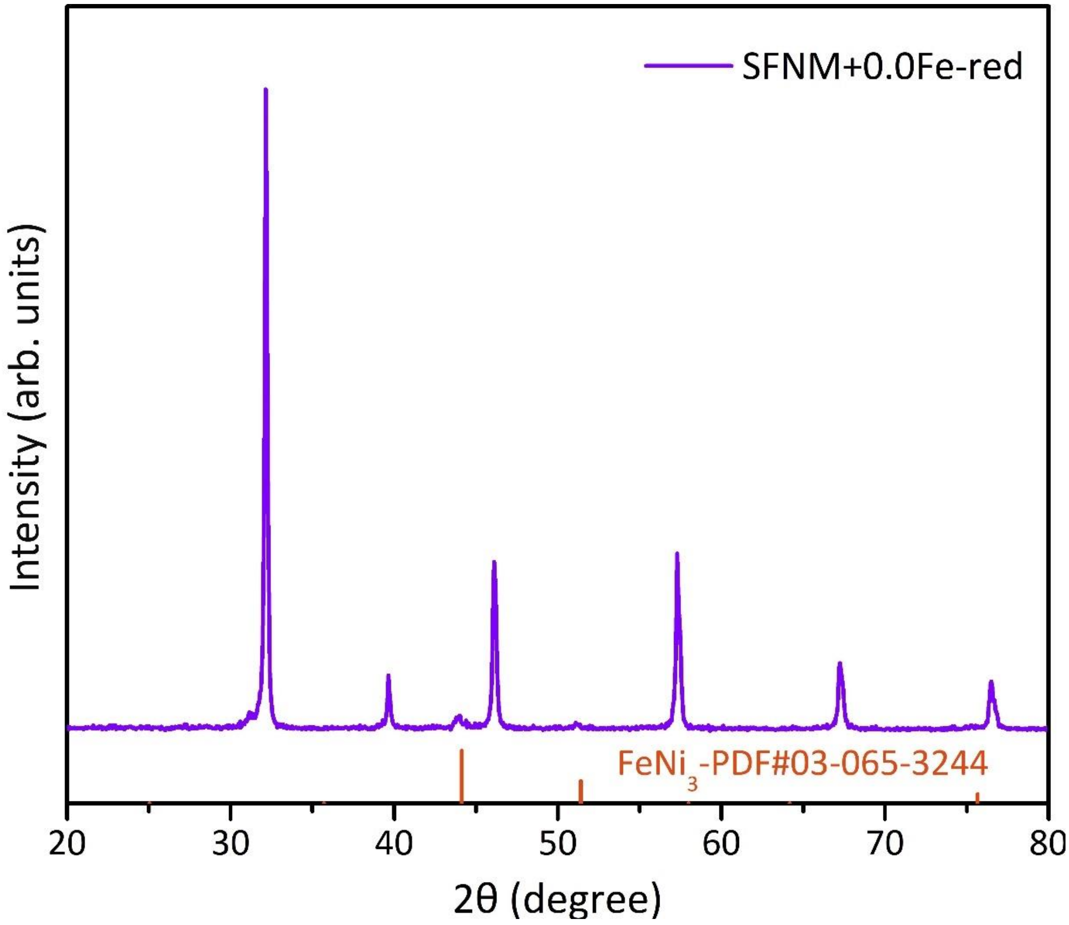

Supplementary Figure 3. XRD pattern of SFNM+0.0Fe-red.

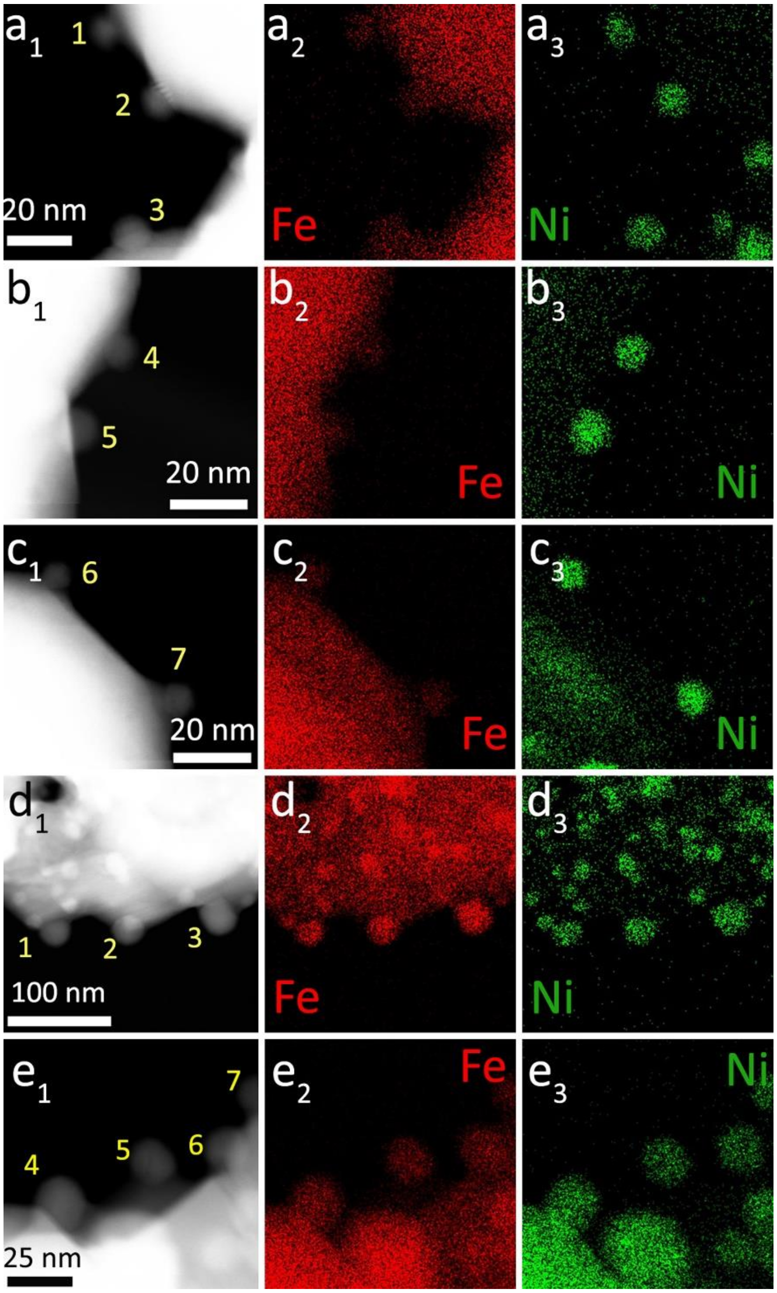

Supplementary Figure 4. STEM with EDS of randomly selected nanoparticles on (**a-c**) SFNM+0.0Fe-red and (**d-e**) SFNM+1.2Fe-red.

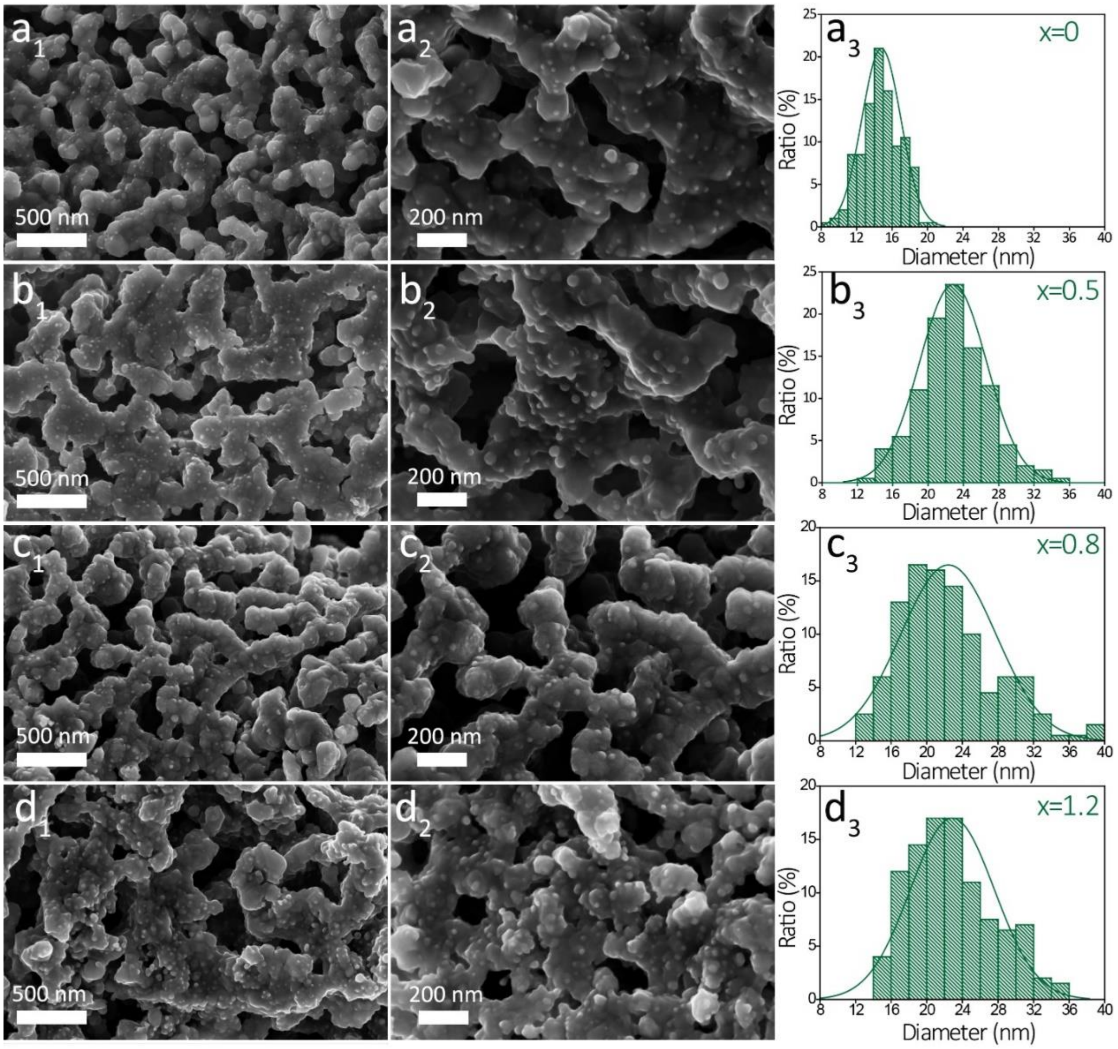

Supplementary Figure 5. (**a_1, a_2-d_1, d_2**) SEM images of microstructures and (**a_3-d_3**) size distribution histograms of the exsolved nanoparticles on SFNM+xFe-red (x=0.0, 0.5, 0.8, 1.2).

To verify the promoted exsolution of Fe-Ni nanoparticles by TIE, the morphologies of SFNM+xFe-red were examined by FE-SEM. As shown in Supplementary Figs. 5a-d, the TIE-assisted procedure yields an average particle size of around 22 nm within a distribution range of 10-40 nm, which

are evidently larger than the corresponded value of 15 nm and 8-22 nm in SFNM+0.0Fe-red. It is interesting to note that the measured diameters of nanoparticles over SFNM+ xFe-red samples (x=0.5, 0.8, 1.2) keep similar, suggesting that increasing the Fe loading would not trigger apparent coarsening of exsolved nanoparticles.

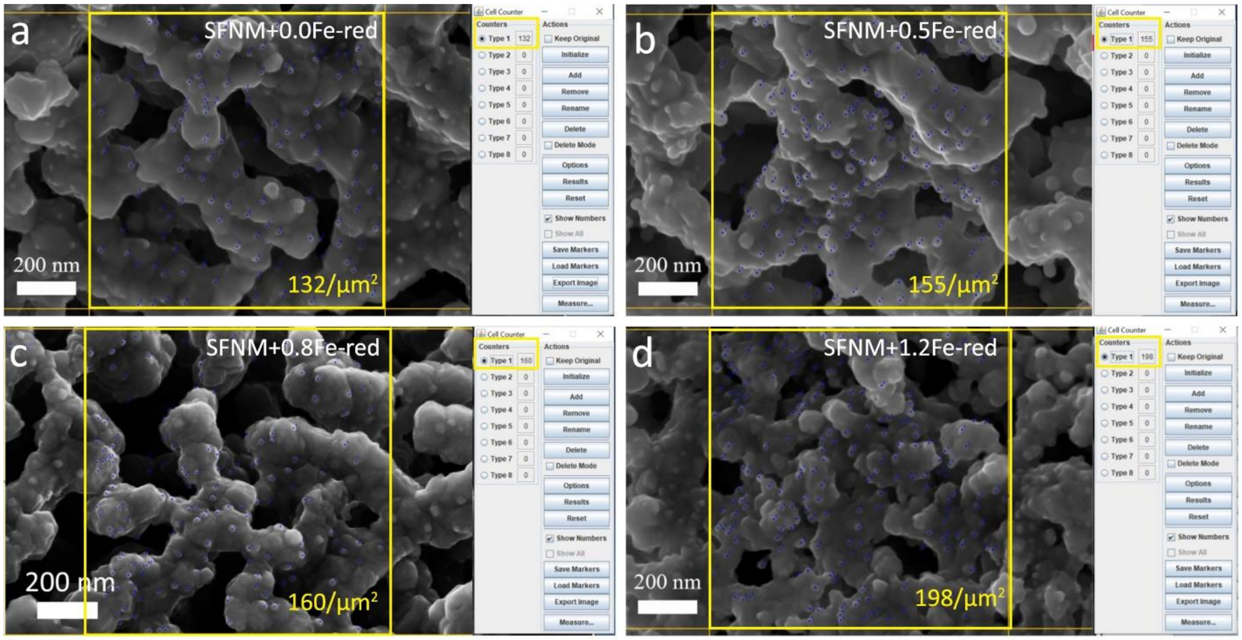

Supplementary Figure 6. Population of exsolved nanoparticles on SFNM+xFe-red (x=0.0, 0.5, 0.8, 1.2) in 1μm^2 area.

Although the size of the exsolved nanoparticles increases by ion exchange, the population of exsolved nanoparticles also increases significantly, thereby expanding the surface-active area. It would lead to the enhanced electrochemical performances.

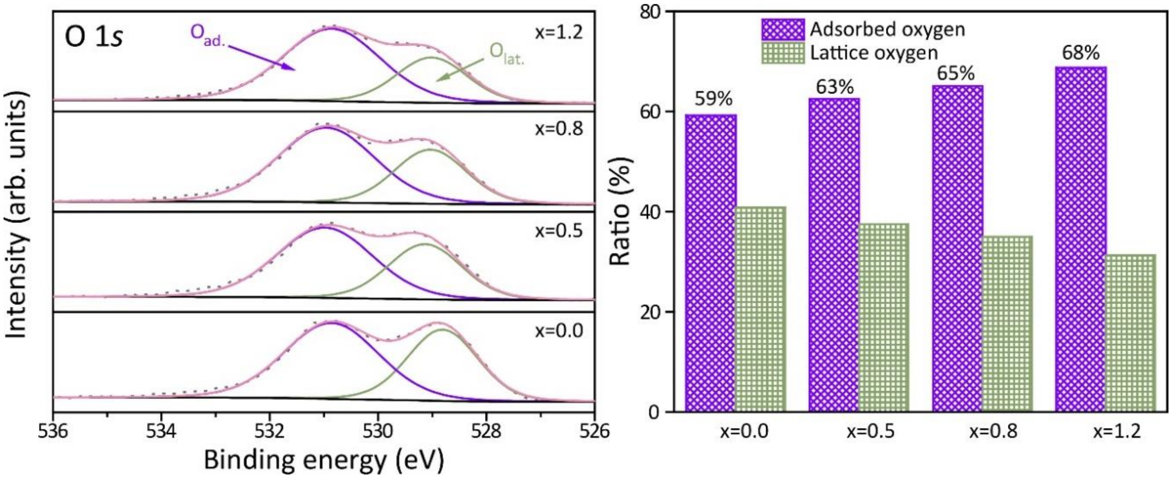

Supplementary Figure 7. XPS O 1*s* spectra for SFNM+xFe-red (x=0.0, 0.5, 0.8, 1.2).

The surface-active oxygen vacancies, together with exsolved nanoparticles, are responsible for accommodating oxygen atom from adsorbed CO_2 molecule and subsequently activating the CO_2[1,2]. Therefore, reactive oxygen concentration on SFNM+xFe-red were inspected by analyzing XPS pattern of O 1*s*. As shown in Supplementary Fig. 7, two distinguishable peaks around 530.0 and 531.6 eV can be attributed to lattice oxygen ($O_{lat.}$) and the adsorbed oxygen ($O_{ad.}$)[3]. The quantitatively analysis by calculating integrated area of the corresponding peak regions shows that there are 59%, 63%, 65% and 69% oxygen vacancies concentration on the surface of SFNM+xFe-red (x=0.0, 0.5, 0.8, 1.2), respectively. Combined with facilitated formation of Fe-Ni alloy nanoparticle, evolution of initial surface

into a more catalytically active surface for SFNM+1.2Fe-red contributes to enhanced CO_2 adsorption and activation.

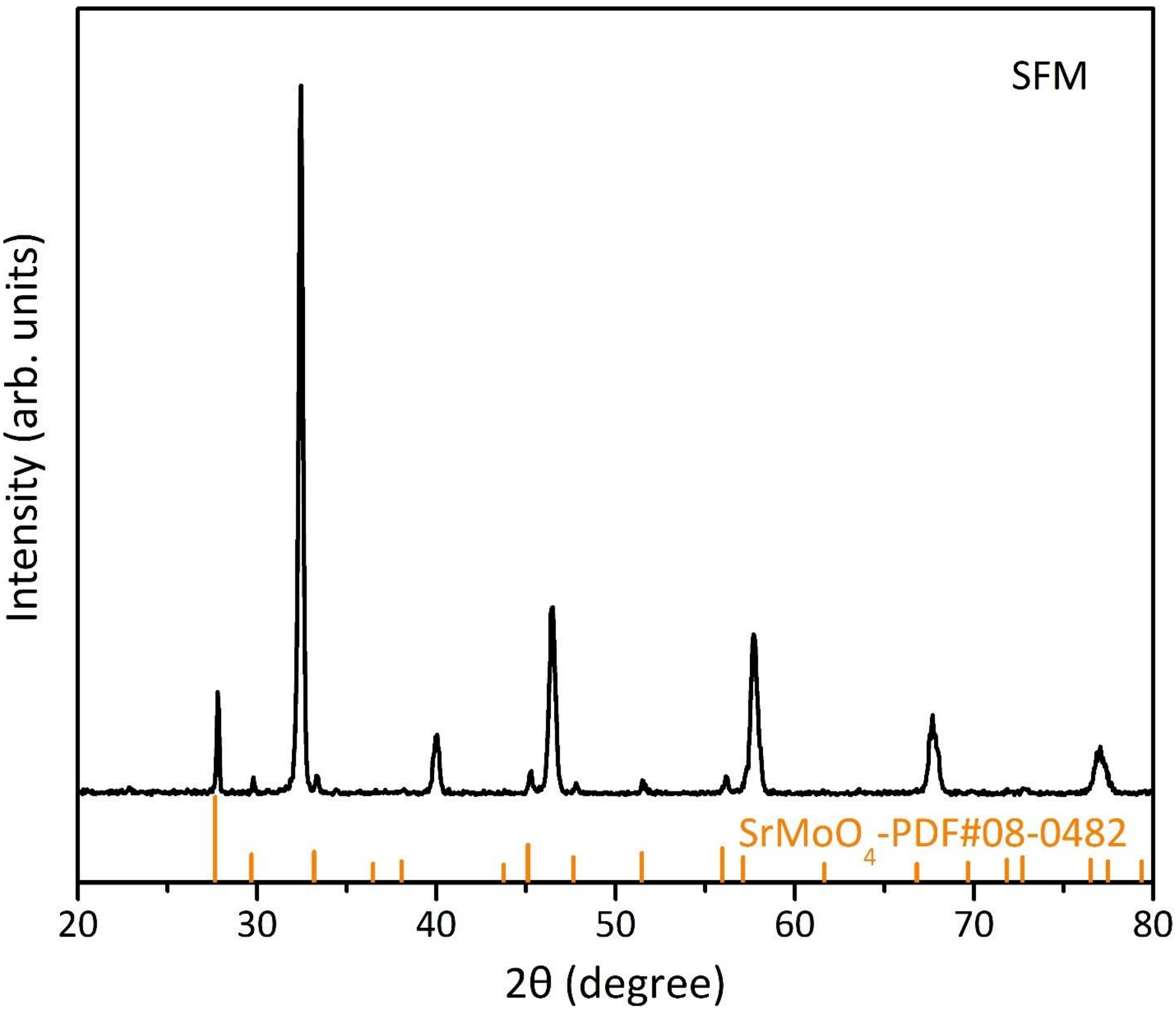

Supplementary Figure 8. XRD of SFM sintered at 1000 ^{o}C in the air for 5 h.

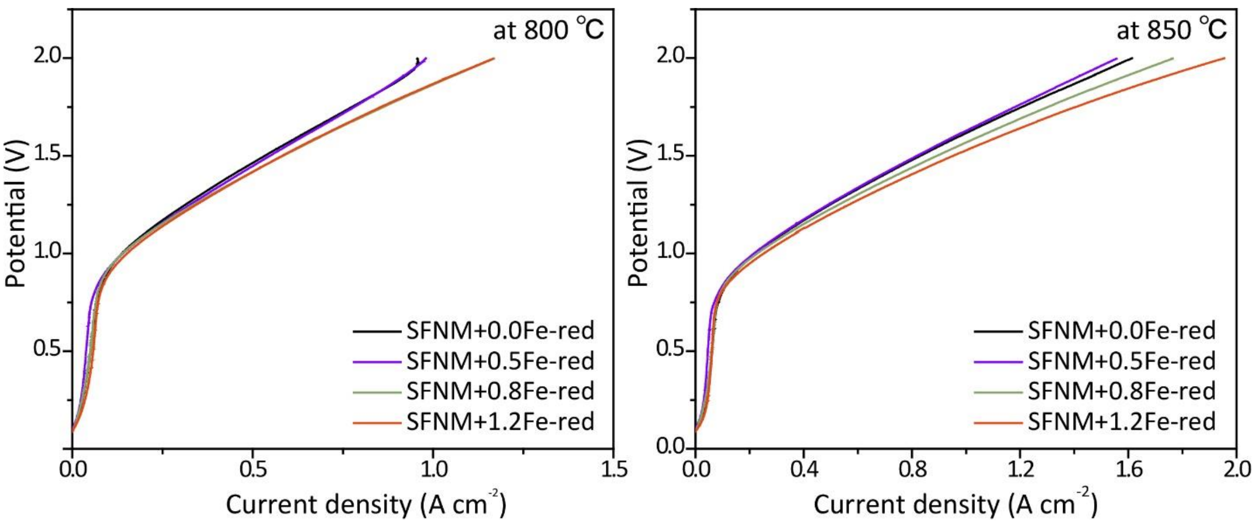

Supplementary Figure 9. I-V curves for SFNM+xFe-red (x=0.0, 0.5, 0.8, 1.2) at 800 and 850 °C.

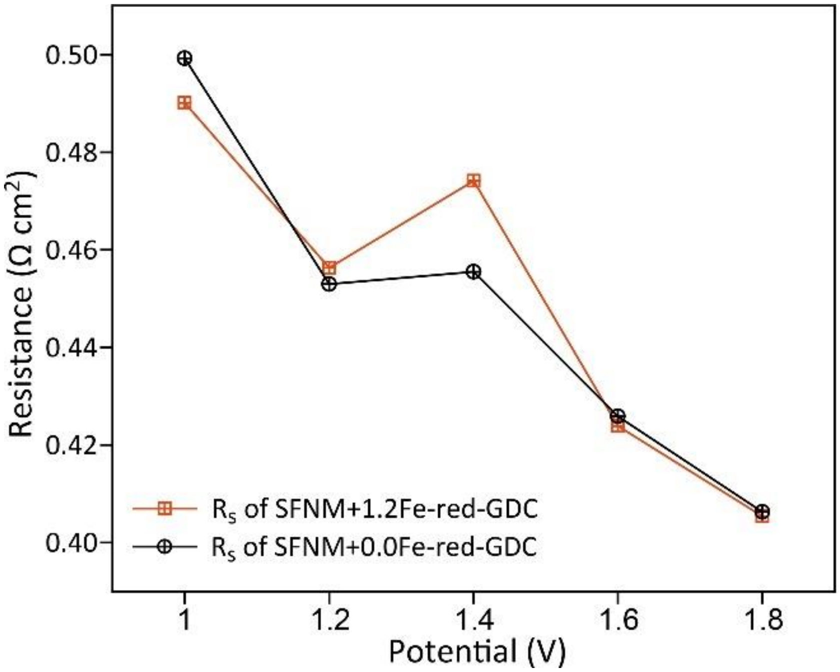

Supplementary Figure 10. Change in R_S of SFNM+0.0Fe-red-GDC and SFNM+1.2Fe-red-GDC with voltages.

From 1 to 1.2 V, the cathode reaction rate accelerates as the voltage increases, leading to an increase of the conductivity, i.e., the oxygen ion transfer resistance is reduced at cathode side and so is R_S. However, the R_S values of both cells increase as the applied voltage increases to 1.4 V, which may be ascribed to the increased resistance at the anode/electrolyte interface. Because the oxygen ions produced at the anode/electrolyte interface gradually accumulate when the cathode reaction kinetics speeds up, this accumulation would hinder the transfer of oxygen ions. This explains why the R_S of the SFNM+1.2Fe-red-GDC with superior oxygen ion conduction at cathode side increases by a larger magnitude than that of

SFNM+0.0Fe-red-GDC. As the applied voltage further increases to 1.6-1.8 V (above the thermally neutral voltage E_H=1.46 V at 850 ^oC[4,5]), the net heat is produced because the entropic heat consumption rate becomes slower than the production rate of the irreversible heat (due to activation, ohmic, and mass transport losses in the electrolyzer[4]). This net heat would cause the promoted transport of oxygen ions, consequently leading to a greatly reduced R_S.

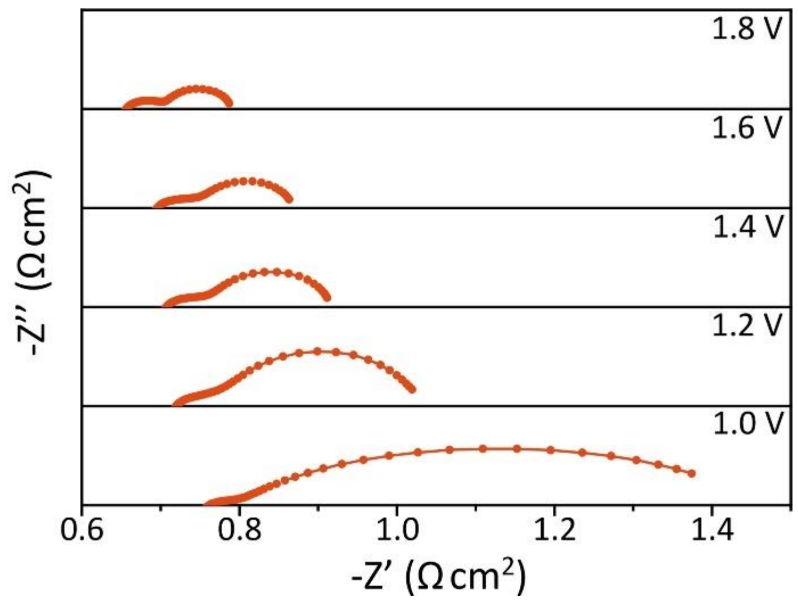

Supplementary Figure 11. Nyquist plots of SFNM+1.2Fe-red-GDC at 800 ^{o}C (Equivalent circuit model: LR($Q_H R_H$)($Q_L R_L$)).

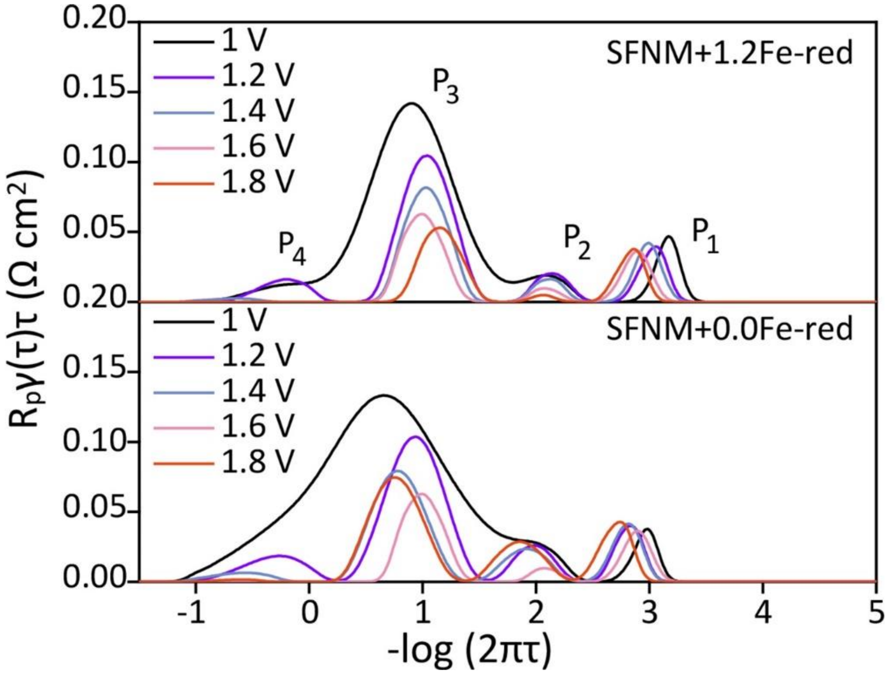

Supplementary Figure 12. Comparison of deconvoluted results of EIS on SFNM+0.0Fe-red and SFNM+1.2Fe-red at applied potentials and 850 ^oC by DRT analysis.

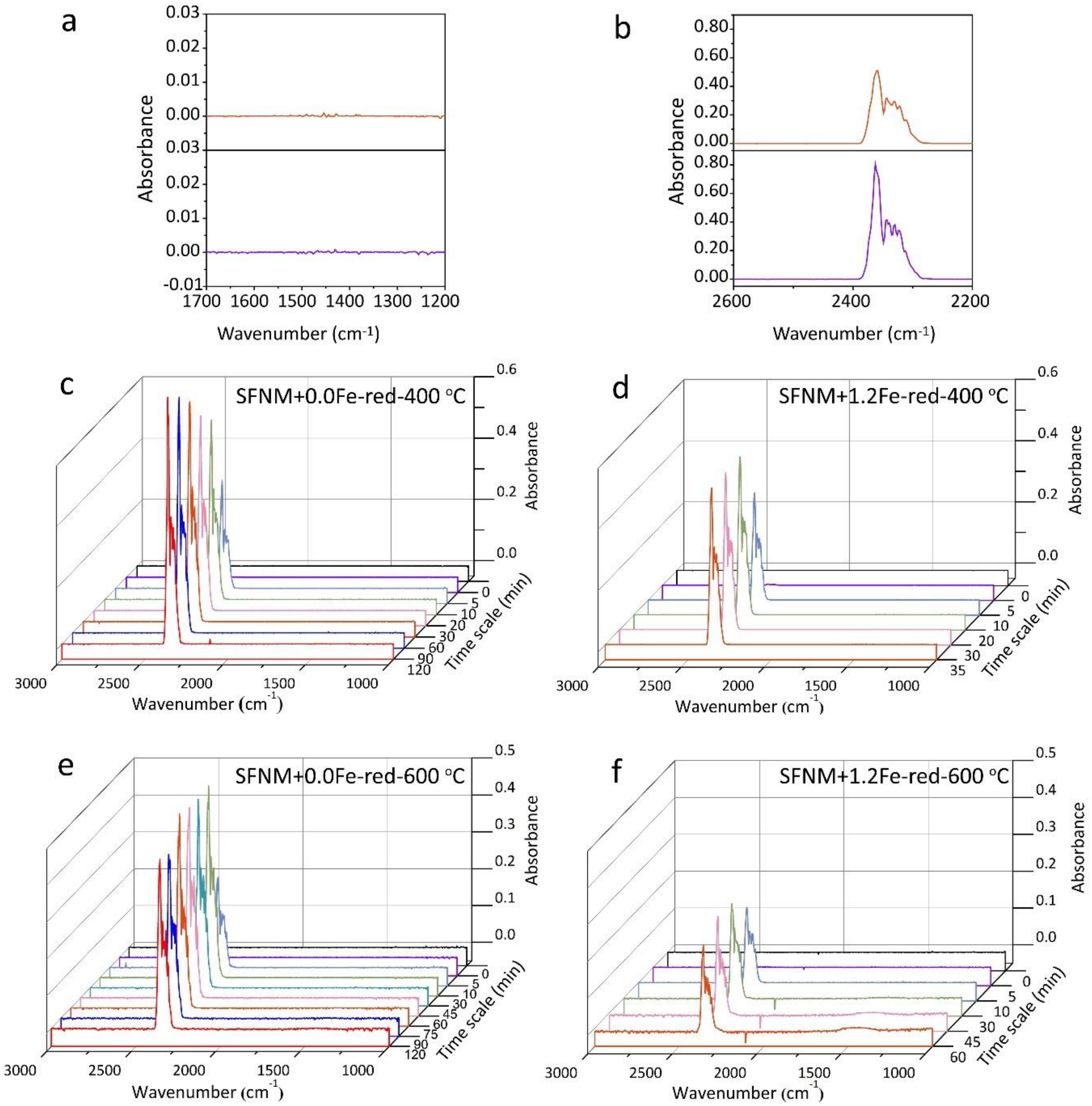

Supplementary Figure 13. FTIR spectra of CO_2 (**a**) chemisorption and (**b**) physisorption for SFNM+0.0Fe-red and SFNM+1.2Fe-red at 400 ^{o}C. Time dimensioned FTIR spectra for (**c, e**) SFNM+0.0Fe-red and (**d, f**) SFNM+1.2Fe-red at 400 and 600 ^{o}C.

To shed light on the synergistic effects of exsolved nanoparticle and surface oxygen vacancy on CO_2 adsorption capacity, SFNM+0.0Fe-red and

SFNM+1.2Fe-red were subjected to FTIR measurement. Supplementary Figs. 13a-b present FTIR patterns of CO_2 adsorbed by SFNM+0.0Fe-red and SFNM+1.2Fe-red at 400 ^oC. Two major infrared band regions can be identified at each temperature, and the bands in range of 2300-2400 cm^{-1} are associated with physical adsorption of CO_2 molecule, whereas those in range of 1300-1550 cm^{-1} correspond to chemisorbed CO_3^{2-} [1,6]. FTIR spectra for both SFNM+0.0Fe-red and SFNM+1.2Fe-red show only physical adsorption of CO_2 at 400 ^oC, yet the higher physical adsorption peaks for SFNM+0.0Fe-red compared to SFNM+1.2Fe-red may be ascribed to its higher surface alkalinity[7]. Raising the temperature to 600 ^oC has led to the chemical adsorption for both samples (Fig. 3d). SFNM+1.2Fe-red has a higher chemical adsorption peak than SFNM+0.0Fe-red after reaching steady stage, suggesting that it has more oxygen storage sites on the surface of support, in agree with the promoted exsolution of Fe-Ni alloy and higher oxygen vacancy concentration on surface. In addition, SFNM+1.2Fe-red exhibits a quick response time to achieve the stable physical/chemical CO_2 adsorption state compared with SFNM+0.0Fe-red (Supplementary Figs. 13c-f), implying the superiority of SFNM+1.2Fe-red in terms of CO_2 adsorption.

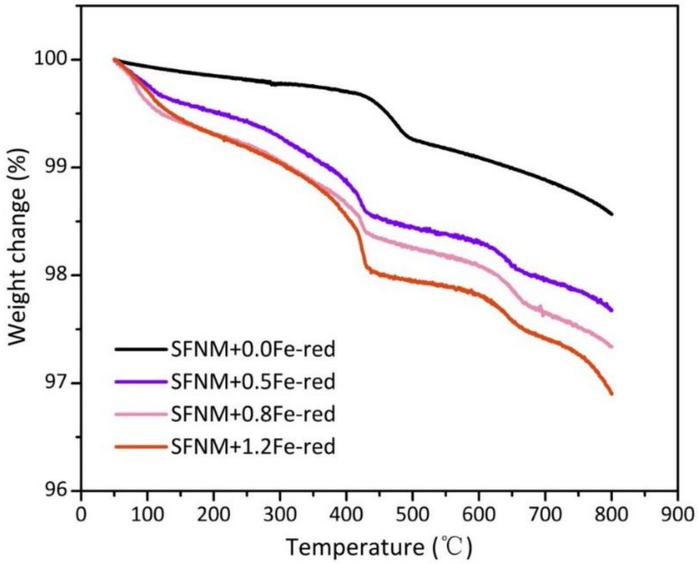

Supplementary Figure 14. TGA of SFNM+xFe-red (x=0.0, 0.5, 0.8, 1.2) in the 5% H_2/95% N_2 atmosphere.

Exsolution of Fe-Ni alloy started with bond-breaking of Fe-O and Ni-O, accompanying with the stripping of lattice oxygen out of perovskite lattice and formation of oxygen vacancies[8]. It is well acknowledged that the oxygen vacancies offer jumping sites for oxygen ion transfer within perovskite lattice. Therefore, the concentration of oxygen vacancies is an important criterion for evaluating the catalytic capacity. TGA measurement was carried out to examine the overall concentration of oxygen vacancy for all reduced samples at high temperature in a reducing (5% H_2/N_2) atmosphere (Supplementary Fig. 14). The gradual weight loss below 400 ^{o}C can be ascribed to the detachment of surface absorbed H_2O molecule[9]. Further raising the temperature induces a continuous decline in sample weight for all

samples, which was attributed to the formation of oxygen vacancy caused by the reduction of B-site cations and exsolution of Fe-Ni alloy, as following equations[10]:

Loss of lattice oxygen caused by reduced oxidation states of Fe and Ni:

$$O_O^X + Fe_{Fe}^X + Ni_{Ni}^X = Fe'_{Fe} + Ni'_{Ni} + \frac{1}{2}O_2 + V_O^{\cdot\cdot}$$

Loss of lattice oxygen caused by exsolution of Fe and Ni:

$$O_O^X + Fe'_{Fe} + Ni'_{Ni} = Fe - Ni + V''_{Fe} + V''_{Ni} + \frac{1}{2}O_2 + V_O^{\cdot\cdot}$$

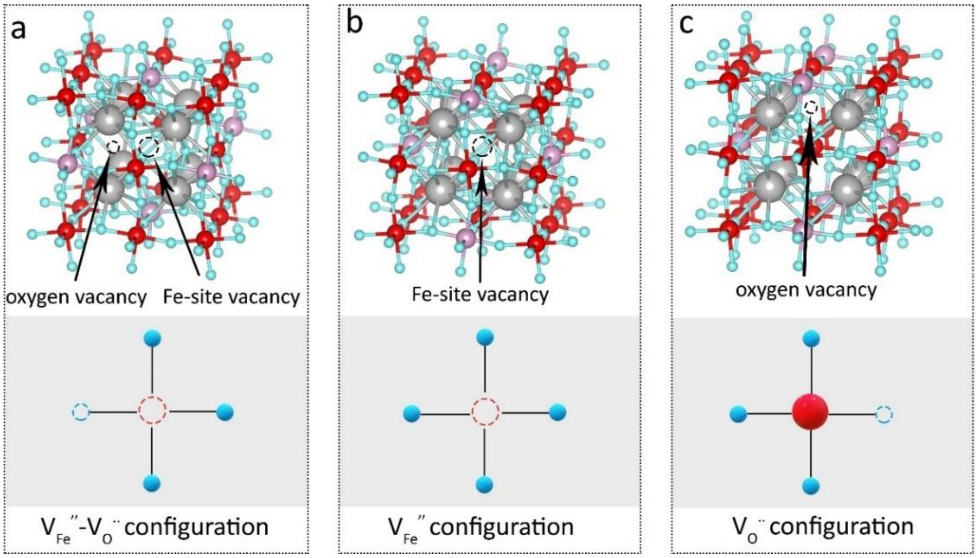

Supplementary Figure 15. Three defective configurations of (a) $V_{Fe}'' - V_O^{\cdot\cdot}$, (b) V_{Fe}'' and (c) $V_O^{\cdot\cdot}$ in SFM. The gray balls represent Sr atoms, the red balls represent Fe atoms, the pink balls represent Mo atoms, the blue balls represent O atoms.

It has been reported that the association (trapping) of oxygen vacancy caused by other defects in perovskite lattice is detrimental to oxygen ion diffusion[11-13]. And it is widely accepted that the association of oxygen vacancy can be determined by calculating the binding energy between the two adjacent point defects[11,14] ($V_{Fe}'' - V_O^{\cdot\cdot}$ pair in our manuscript). The binding energy (E_{bind}) can be calculated by following equation[12]:

$$E_{bind} = E_{defects\,pair} - \left(\sum_{component} E_{isolated\,defect}\right)$$

In our case,

$$E_{bind} = E_{(V_{Fe}'' - V_{O}^{\cdot\cdot})} - (E_{(V_{Fe}'')} + E_{(V_{O}^{\cdot\cdot})})$$

To simplify the model, we assume that all Ni elements are exsolved from the SFNM substrate after the reduction. Therefore, the binding energy between the Fe-site vacancy and the oxygen vacancy is calculated in the $Sr_2Fe_{1.5}Mo_{0.5}O_6$ (SFM). The three defective configurations and the corresponding formation energy are provided in Supplementary Fig. 15 and Supplementary Table 5. As a result, the calculated binding energy of $V_{Fe}'' - V_{O}^{\cdot\cdot}$ is -1.73 eV. It indicates that the existence of B-site vacancies would hinder the transport of oxygen vacancies, thus resulting in the reduction of the free oxygen vacancy population.

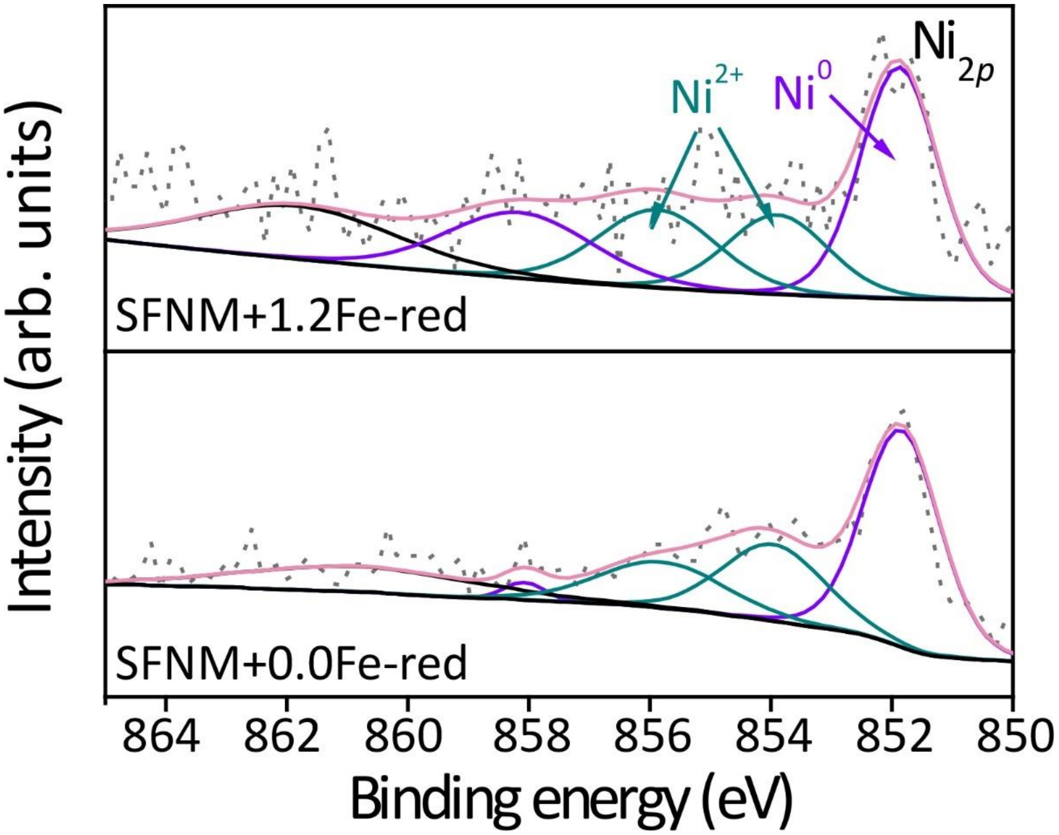

Supplementary Figure 16. XPS of Ni 2*p* for SFNM+0.0Fe-red and SFNM+1.2Fe-red.

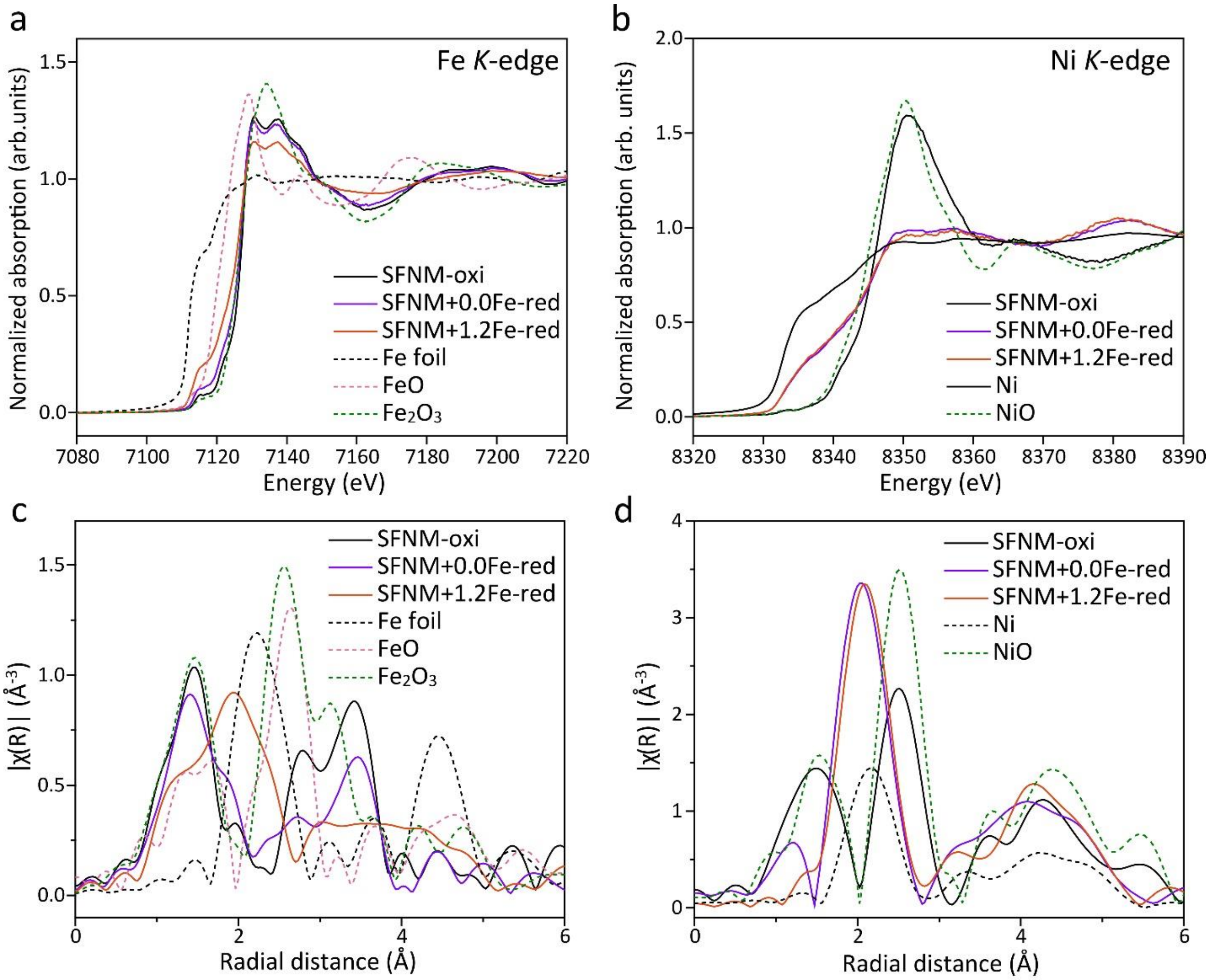

Supplementary Figure 17. (a) Fe K-edge and (b) Ni K-edge X-ray absorption near-edge structure (XANES) spectra of SFNM-oxi, SFNM+0.0Fe-red and SFNM+1.2Fe-red, together with reference samples Fe-foil, FeO, Fe_2O_3, Ni-foil, and NiO. Fourier-transformed (c) Fe K-edge and (d) Ni K-edge extended X-ray absorption fine structure (EXAFS) spectra of SFNM-oxi, SFNM+0.0Fe-red and SFNM+1.2Fe-red, together with reference samples Fe-foil, FeO, Fe_2O_3, Ni-foil, and NiO.

The Fe and Ni K-edge X-ray absorption near edge structure (XANES) spectra along with the extended X-ray absorption fine structure (EXAFS) spectra among air-sintered SFNM-oxi, SFNM+0.0Fe-red and SFNM+1.2Fe-

red are provided in Supplementary Fig. 17[15,16]. The shift of pre-edge of Fe and Ni K-edge for SFNM+0.0Fe-red and SFNM+1.2Fe-red to lower energy with respect to that for SFNM-oxi is associated with the decrease of valence states of Fe and Ni after the reduction[17,18]. It is clear that the valence states of Ni are almost identical in SFNM+0.0Fe-red and SFNM+1.2Fe-red (the peaks of SFNM+0.0Fe-red and SFNM+1.2Fe-red overlap with those of Ni foil and NiO in the EXAFS spectra and the Ni peaks of both reduced samples are similar), while the Fe valence state for SFNM+1.2Fe-red is apparently lower (the overlapping area between the peak of SFNM+1.2Fe-red and the peak of Fe foil is much larger than that between SFNM+0.0Fe-red and Fe foil in the EXAFS spectra), which is consistent with the Fe and Ni 2*p* XPS spectra. It suggests that the amount of the Ni exsolution has almost reached the peak value via conventional exsolution due to its lower co-segregation energy. The lower Fe valence state of SFNM+1.2Fe-red can be ascribed to the participation of external Fe sources in the formation of Fe-Ni alloy nanoparticles.

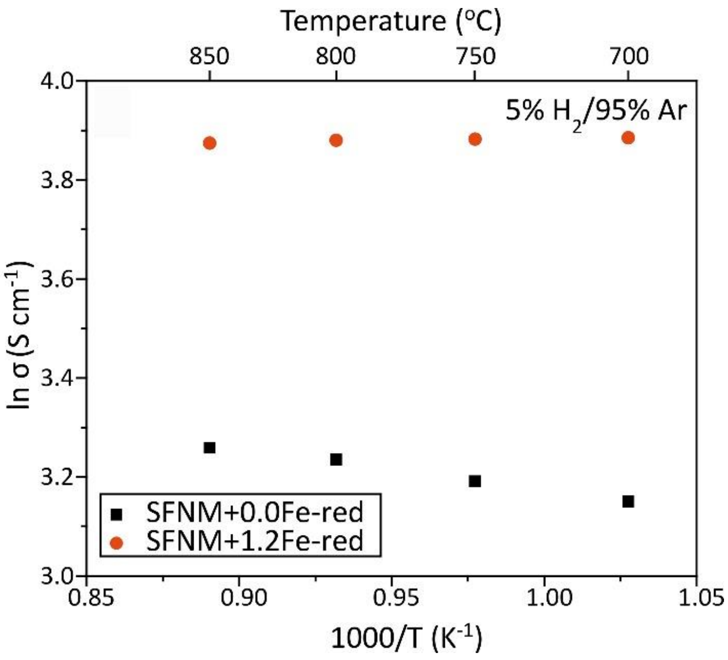

Supplementary Figure 18. Temperature-dependent electrical conductivities of SFNM+0.0Fe-red and SFNM+1.2Fe-red under 5% H_2/95% Ar atmosphere.

As depicted in Supplementary Fig. 18, the electrical conductivities of SFNM+0.0Fe-red and SFNM+1.2Fe-red in the 5% H_2/95% Ar atmosphere at 850 ^{o}C reach 26.0 and 48.2 S cm^{-1}, respectively, which are higher than those t in the 50% CO_2/50% CO atmosphere. It can be ascribed to the contribution of the more exsolved Fe-Ni alloy nanoparticles formed under the more reducing condition in addition to the conduction pathways in perovskite scaffold[19]. A large number of metallic nanoparticles on SFNM+1.2Fe-red leads to the slightly reduced conductivity as the temperature increases in the 5% H_2/95% Ar atmosphere.

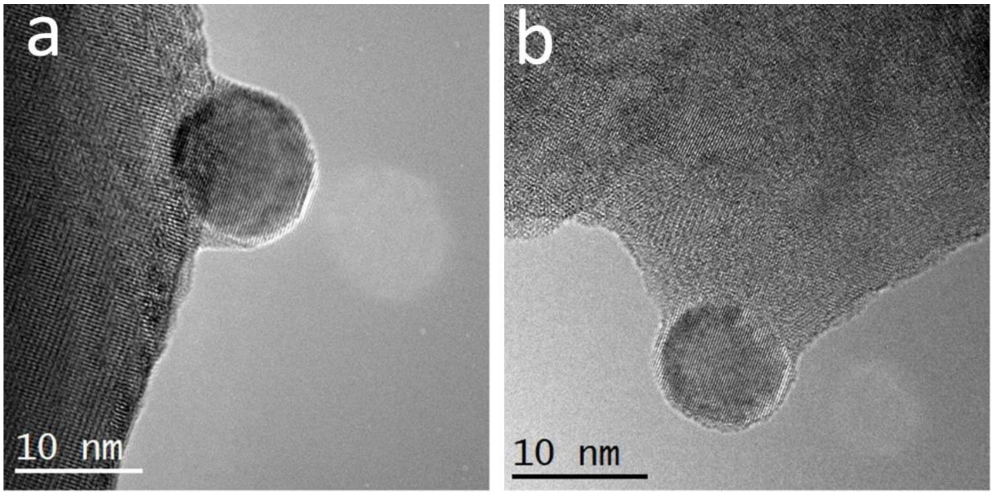

Supplementary Figure 19. High resolution TEM results for (**a**) SFNM+0.0Fe-red and (**b**) SFNM+1.2Fe-red.

High-resolution TEM of individual particle shows that the exsolved nanosphere with TIE assistance remains locked closely at the surface of matrix, maintaining the key virtues of conventionally exsolution-induced socked interface, which helps anchor the active sites[20] (Supplementary Figs. 19a-b).

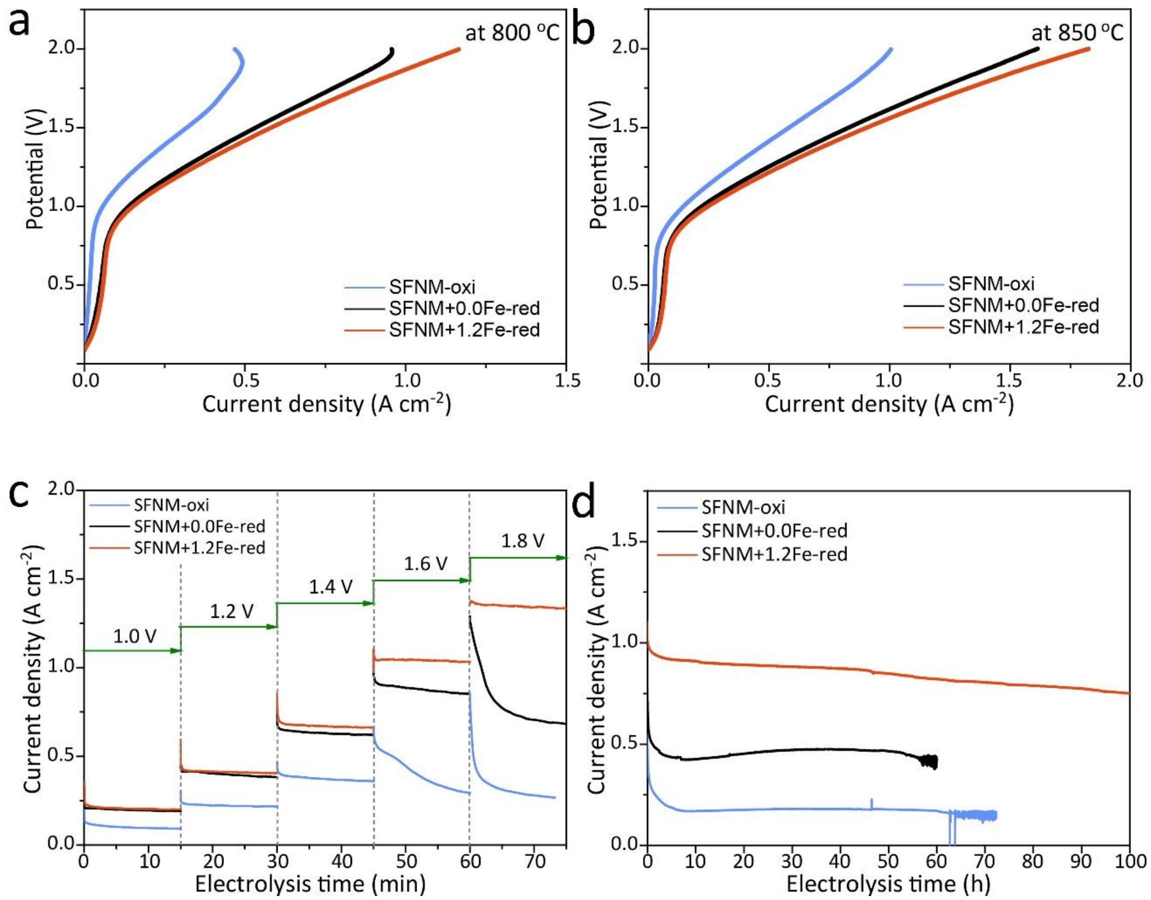

Supplementary Figure 20. Comparison of electrochemical performance of SFNM-oxi-GDC with that of SFNM+0.0Fe-red-GDC and SFNM+1.2Fe-red-GDC. I-V curves of SFNM-oxi-GDC with that of SFNM+0.0Fe-red-GDC and SFNM+1.2Fe-red-GDC at (**a**) 800 °C and (**b**) 850 °C. Comparison of (**c**) Short-term stability and (**d**) long-term stability performances at 850 °C of SFNM-oxi-GDC with that of SFNM+0.0Fe-red-GDC and SFNM+1.2Fe-red-GDC. (Note: the break point at 47 h in Supplementary Fig. 20d is caused by updating the CO_2 cylinder.)

To further clarify the degradation by exsolution, the catalytic activity and stability performances of $Sr_2Fe_{1.3}Ni_{0.2}Mo_{0.5}O_6$ (SFNM) based SOEC without

exsolution have been evaluated for comparison (Supplementary Fig. 20). As can be seen from the current density-voltage (j-V) curves at 800 and 850 ^{o}C, SFNM-based SOEC without exsolution (SFNM-oxi-GDC) exhibits the lower j than the j values of the SFNM+0.0Fe-red-GDC and SFNM+1.2Fe-red-GDC under the same condition. Additionally, the SFNM-oxi-GDC shows the similar short-term/long-term stability profiles as the SFNM+0.0Fe-red-GDC. In term of the short-term stability, the SFNM-oxi-GDC shows satisfactory stability at 1.0-1.4 V, while the SOEC experiences a rapid degradation when the voltage exceeds 1.6 V. For the long-term stability, the SOEC experiences a significant degradation at the initial stage, followed by a steady j-V profile. In fact, the j also experiences a slight increase, peaking at 31 h with a maximum j of 0.18 A cm^{-2}. Then, the SFNM-oxi-GDC shows a visible degradation after 60 h electrolysis, and finally destabilized.

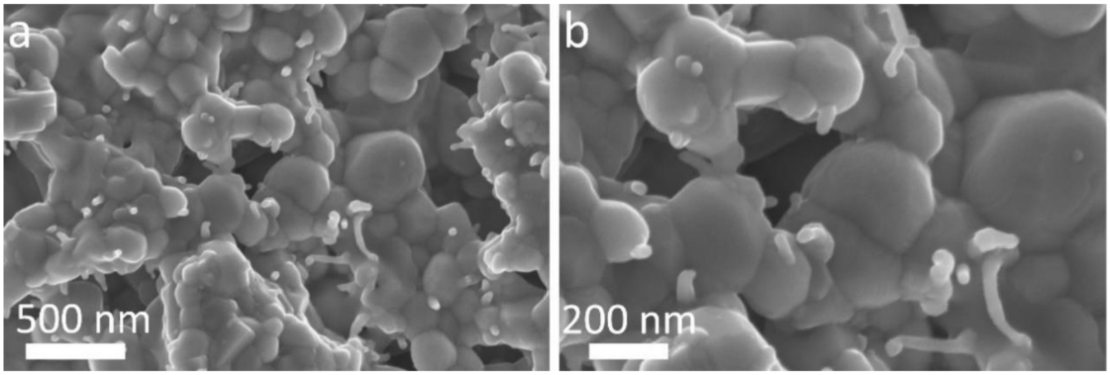

Supplementary Figure 21. SEM images of cathode surface microstructures of SFNM-oxi-GDC after long-term stability at 1.6 V and 850 ^{o}C.

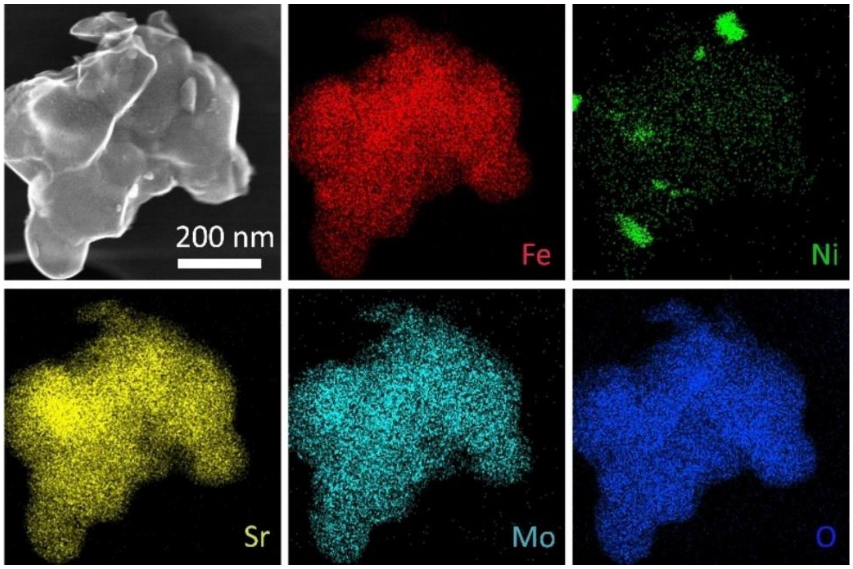

Supplementary Figure 22. TEM images of cathode surface microstructures of SFNM-oxi-GDC after long-term stability at 1.6 V and 850 ^{o}C.

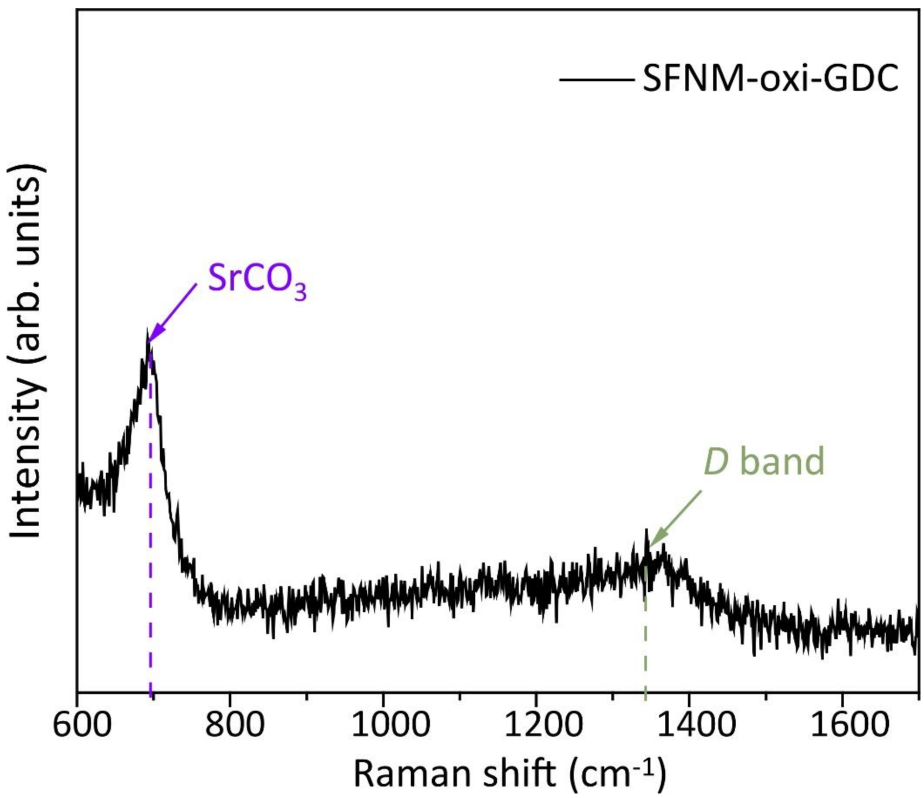

Supplementary Figure 23. Raman spectrum collected from cathode surface of SFNM-oxi-GDC after the long-term stability test.

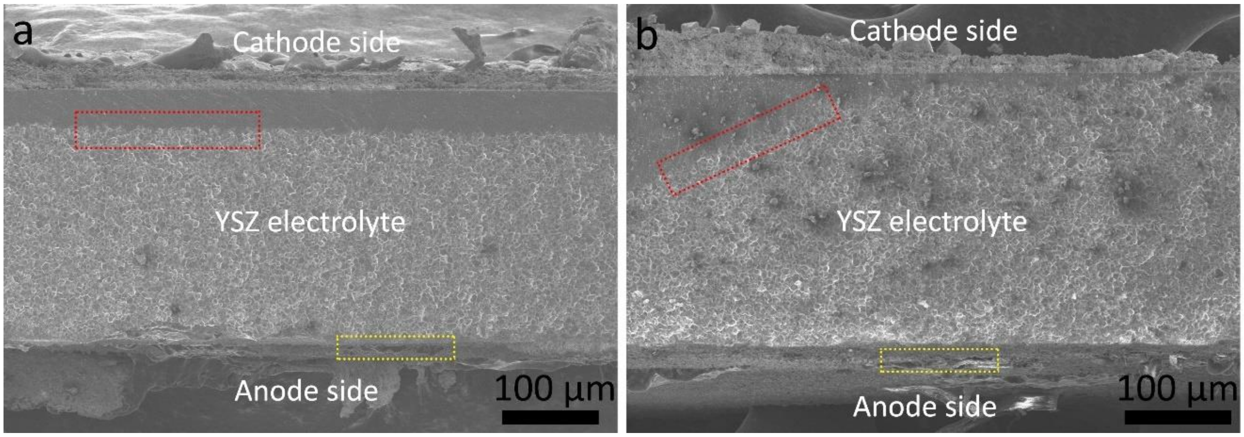

Supplementary Figure 24. SEM images of cross section of (a) SFNM+0.0Fe-red-GDC and (b) SFNM+1.2Fe-red-GDC after long-term stability tests at 1.6 V and 850 ^{o}C.

The degradations of the electrolyte and the anode under the harsh conditions are analyzed[21]. Supplementary Fig. 24 shows the SEM images of the cross sections of SFNM+0.0Fe-red-GDC and SFNM+1.2Fe-red-GDC after the long-term stability tests at 1.6 V and 850 ^{o}C. The significant grain coarsening of YSZ electrolyte can be observed near the anode|GDC|YSZ interfaces of both cells, and this grain growth propagates along the electrolyte and progresses towards the cathode side. Furthermore, the pore formation near the anode|GDC|YSZ interfaces and even the delamination of anode from the electrolyte can be observed, which is supposed to originate from the high oxygen partial pressures formed at the interfaces during the long-term CO_2 electrolysis at high voltage.

Supplementary Table 1. Fe and Ni content in randomly selected nanoparticles on SFNM+0.0Fe-red and SFNM+1.2Fe-red.

**Exsolved nanoparticles**	**Atom ratio (Fe/Ni, Ni as reference that Ni=1)**	
Number	**SFNM+0.0Fe-red**	**SFNM+1.2Fe-red**
Site 1	0.36	3.05
Site 2	0.34	2.90
Site 3	0.32	3.45
Site 4	0.36	1.98
Site 5	0.44	2.27
Site 6	0.41	2.65
Site 7	0.35	2.70
**Average**	**0.37**	**2.71**

Supplementary Table 2. EIS fitting values (R_S, R_H, R_L) of SFNM+0.0Fe-red-GDC and SFNM+1.2Fe-red-GDC at applied voltages.

Potential (V)	Samples	R_s (Ω cm^2)	R_H (Ω cm^2)	R_L (Ω cm^2)	R_p (Ω cm^2)
1	SFNM+0.0Fe-red	0.4992	0.01906	0.5066	0.52566
	SFNM+1.2Fe-red	0.4901	0.3306	0.04527	0.37587
1.2	SFNM+0.0Fe-red	0.453	0.09258	0.1726	0.26518
	SFNM+1.2Fe-red	0.4563	0.0974	0.1479	0.2453
1.4	SFNM+0.0Fe-red	0.4555	0.07313	0.1142	0.18733
	SFNM+1.2Fe-red	0.4741	0.07575	0.07375	0.1495
1.6	SFNM+0.0Fe-red	0.4259	0.05786	0.08628	0.14414
	SFNM+1.2Fe-red	0.4239	0.05847	0.04617	0.10464
1.8	SFNM+0.0Fe-red	0.4063	0.07069	0.1046	0.17529
	SFNM+1.2Fe-red	0.4054	0.04964	0.03924	0.08888

Supplementary Table 3. EIS fitting values (R_S, R_H, R_L) of SFNM+1.2Fe-red-GDC at 800 ^oC (Equivalent circuit model: $LR(Q_HR_H)(Q_LR_L)$).

Potential (V)	R_s (Ω cm^2)	R_H (Ω cm^2)	R_L (Ω cm^2)	R_P (Ω cm^2)
1	0.7375	0.0714	0.6480	0.7194
1.2	0.6901	0.1796	0.1871	0.3667
1.4	0.6847	0.1254	0.1090	0.2344
1.6	0.6743	0.0910	0.1064	0.1974
1.8	0.6441	0.0751	0.0700	0.1451

Supplementary Table 4. Comparison of the current density, polarization resistance, CO productivity with other state-of-art exsolved SOECs for CO_2 electrocatalysis.

**Perovskite cathode (Exsolved nanoparticles)**	**Electrolyte| Anode**	**Current density at 1.6 V (A cm^{-2})**	**Polarization resistance (Ω cm^2)**	**CO productivity (mL min^{-1} cm^{-2})**	**Ref.**
**SFNM+1.2Fe-red (Fe-Ni alloy)**	**YSZ|LSCF-GDC**	**1.12 (850 ºC)**	**0.14/0.10/0.08 (1.4/1.6/1.8 V, 850 ºC) 0.23/0.19/0.14 (1.4/1.6/1.8 V, 800 ºC)**	**6.43 (1.6 V and 850 ºC)**	**This work**
$Sr_2Fe_{1.58}Mo_{0.5}O_{6-\delta}$ (Metallic Fe)	LSGM| LSM-SDC	0.95 (850 ºC)	0.29 (1.8 V and 850 ºC)	-	[22]
$(La_{0.2}Sr_{0.8})_{0.85}Ti_{0.8}Cr_{0.1}Ni_{0.1}O_{3-\delta}$ (Metallic Ni)	LSGM| LSM-SDC	0.68 (850 ºC)	0.43 (1.6 V and 850 ºC)	3.27 (1.6 V and 850 ºC)	[23]
$Sr_2Fe_{1.35}Mo_{0.45}Co_{0.2}O_{6-\delta}$ (Co-Fe alloy)	LSGMI BSCF-GDC	1.20 (800 ºC)	0.28 (1.6 V and 800 ºC)	ca. 8.50 (1.6 V and 800 ºC)	[24]
$(La_{0.2}Sr_{0.8})_{0.95}Ti_{0.85}Mn_{0.1}Ni_{0.05}O_{3-\delta}$ (Metallic Ni)	YSZ|LSM	0.54 (800 ºC)	0.51 (1.6 V and 800 ºC)	3.67 (1.6 V and 800 ºC)	[2]
$Sr_2Fe_{1.35}Mo_{0.45}Ni_{0.2}O_{6-\delta}$ ($FeNi_3$ alloy)	LSGM| LSCF-GDC	0.93 (800 ºC)	0.20 (1.6 V and 800 ºC)	ca. 7.50 (1.6 V and 800 ºC)	[25]
$La_{1.2}Sr_{0.8}Mn_{0.4}Fe_{0.6}O_{4-\delta}$ (Metallic Fe)	LSGM| LSCF−GDC	1.43 (1.5 V, 800 ºC)	0.326 (1.5 V and 800 ºC)	-	[26]
$La_{0.6}Ca_{0.4}Fe_{0.8}Ni_{0.2}O_{3-\delta}$ (Fe-Ni alloy)	YSZ|$La_{0.6}Ca_{0.4}Fe_{0.8}Ni_{0.2}O_{3-\delta}$	1.18 (800 ºC)	0.399 (1.3 V and 800 ºC)	-	[27]
$Sr_2Fe_{1.25}Cu_{0.25}Mo_{0.5}O_{6-\delta}$ (Fe-Cu alloy)	LSGM| LSCF−SDC	5.40 (850 ºC)	0.46 (ca. 0.92 V and 850 ºC)	12.80 (1.4 V and 800 ºC)	[28]

Note: Some values of the current density at 1.6 V of the cited literatures are determined by the Digitizer function of Origin software.

Supplementary Table 5. The corresponded energies of three defective configurations and perfect SFM.

Configuration	Energy (eV)
$V_{Fe}'' - V_{O}^{\cdot\cdot}$ in SFM	-258.45
V_{Fe}'' in SFM	-262.85
$V_{O}^{\cdot\cdot}$ in SFM	-268.81
Perfect SFM	-274.94

Table Supplementary Table 6. Comparison of the short-term stability with the state-of-art P-eNs for pure CO_2 electrocatalysis in SOEC.

Perovskite cathode (Exsolved nanoparticles)	Current density loss rate at 1.6 V (A cm^{-2} min^{-1})	Current density loss rate at 1.8 V (A cm^{-2} min^{-1})	Ref.
**SFNM+1.2Fe-red (Fe-Ni alloy)**	**0.003 (850 °C, 15 min)**	**0.001 (850 °C, 15 min)**	**This work**
**SFNM+0.0Fe-red (Fe-Ni alloy)**	**0.008 (850 °C, 15 min)**	**0.040 (850 °C, 15 min)**	**This work**
$Sr_2Fe_{1.58}Mo_{0.5}O_{6-\delta}$ (Metallic Fe)	0.002 (850 °C, 15 min)	0.002 (850 °C, 15 min)	[22]
$(La_{0.65}Sr_{0.3}Ce_{0.05})_{0.9}(Cr_{0.5}Fe_{0.5})_{0.85}Ni_{0.15}O_{3-\delta}$ (Fe-Ni alloy)	0.022 (850 °C, 15 min)	0.030 (850 °C, 15 min)	[29]
$Sr_2Fe_{1.35}Mo_{0.45}Ni_{0.2}O_{6-\delta}$ ($FeNi_3$ alloy)	0.005 (800 °C, 20 min)	-	[25]
$(La_{0.2}Sr_{0.8})_{0.95}Ti_{0.85}Mn_{0.1}Ni_{0.05}O_{3-\delta}$ (Metallic Ni)	0.004 (800 °C, 15 min)	0.005 (800 °C, 15 min)	[2]

Note: The values of the current density in the short-term stability of the cited literatures are determined by the Digitizer function of Origin software.

The short-term stability performances of SFNM+0.0Fe-red-GDC and SFNM+1.2Fe-red-GDC are compared with those of other state-of-art P-eNs-based SOECs, as shown in Supplementary Table 6. In particular, $Sr_2Fe_{1.58}Mo_{0.5}O_{6-\delta}$ with the exsolved Fe nanoparticles in reference 27 shows the comparable stability to SFNM+1.2Fe-red-GDC, which can be attributed to the super-stoichiometric Fe occupation at the B-site. It leads to the

sufficient B-site occupation and robust perovskite structure after the reduction, which is consistent with our experimental results and the B-site supplement mechanism proposed in this work.

References

1. Deka, D. J. *et al.* Investigation of hetero-phases grown via in-situ exsolution on a Ni-doped (La,Sr)FeO_3 cathode and the resultant activity enhancement in CO_2 reduction. *Appl. Catal. B* **286**, 119917 (2021).
2. Ye, L. *et al.* Enhancing CO_2 electrolysis through synergistic control of non-stoichiometry and doping to tune cathode surface structures. *Nat. Commun.* **8**, 14785 (2017).
3. Zhi, M. *et al.* Single crystalline $La_{0.5}Sr_{0.5}MnO_3$ microcubes as cathode of solid oxidefuel cell. *Energy Environ. Sci.* **4**, 139-144 (2011).
4. O'hayre, R., Cha, S.-W., Colella, W. & Prinz, F. B. *Fuel cell fundamentals*. (John Wiley & Sons, 2016).
5. Park, S. *et al.* In situ exsolved Co nanoparticles on Ruddlesden-Popper material as highly active catalyst for CO_2 electrolysis to CO. *Appl. Catal. B* **248**, 147-156 (2019).
6. Xi, X. *et al.* Reducing d-p band coupling to enhance CO_2 electrocatalytic activity by Mg-doping in $Sr_2FeMoO_{6-\delta}$ double perovskite. *Nano Energy* **82**, 105707 (2020).
7. Neagu, D., Tsekouras, G., Miller, D. N., Menard, H. & Irvine, J. T. In situ growth of nanoparticles through control of non-stoichiometry. *Nat. Chem.* **5**, 916-923 (2013).
8. Kwon, O. *et al.* Exsolution trends and co-segregation aspects of self-grown catalyst nanoparticles in perovskites. *Nat. Commun.* **8**, 15967 (2017).
9. Liu, S., Liu, Q. & Luo, J.-L. Highly stable and efficient catalyst with in situ exsolved Fe–Ni alloy nanospheres socketed on an oxygen deficient perovskite for direct CO_2 electrolysis. *ACS Catal.* **6**, 6219-6228 (2016).
10. Hou, N. *et al.* A-Site Ordered Double Perovskite with in Situ Exsolved Core–Shell Nanoparticles as Anode for Solid Oxide Fuel Cells. *ACS Appl. Mater. Interfaces* **11**, 6995-7005 (2019).
11. Islam, M. S. & Davies, R. A. Atomistic study of dopant site-selectivity and defect association in the lanthanum gallate perovskite. *J. Mater. Chem.* **14**, 86-93 (2004).
12. Islam, A. J. M. S. Atomic-scale insight into $LaFeO_3$ perovskite defect nanoclusters and ion migration. *J Phys. Chem. C* **112**, 4455–4462 (2008).
13. Neagu, D. & Irvine, J. T. S. Enhancing electronic conductivity in strontium titanates through correlated A and B-site doping. *Chem. Mater.* **23**, 1607-1617 (2011).

14. Mogensen, M., Lybye, D., Bonanos, N., Hendriksen, P. & Poulsen, F. Factors controlling the oxide ion conductivity of fluorite and perovskite structured oxides. *Solid State Ion.* **174**, 279-286 (2004).
15. Ravel, B. & Newville, M. ATHENA, ARTEMIS, HEPHAESTUS: data analysis for X-ray absorption spectroscopy using IFEFFIT. *J. Synchrotron Rad.* **12**, 537-541 (2005).
16. D'Orazio, A. C. *et al.* High temperature X-ray absorption spectroscopy of the local electronic structure and oxide vacancy formation in the $Sr_2Fe_{1.5}Mo_{0.5}O_{6-\delta}$ solid oxide fuel cell anode catalyst. *ACS Appl. Energy Mater.* **2**, 3061-3070 (2019).
17. Oishi, M., Sakuragi, T., Ina, T., Oshima, N. & Fujishiro, F. In situ evaluation of the electronic/local structure in B-site mixed perovskite-type oxide $SrFe_{0.6}Mn_{0.4}O_{3-\delta}$. *J. Solid State Chem.* **294**, 121893 (2021).
18. Park, S. *et al.* Ruddlesden–Popper oxide $(La_{0.6}Sr_{0.4})_2(Co,Fe)O_4$ with exsolved CoFe nanoparticles for a solid oxide fuel cell anode catalyst. *Energy Technol.* **9**, 2100116 (2021).
19. Yang, X. *et al.* Enhancing stability and catalytic activity by in situ exsolution for high-performance direct hydrocarbon solid oxide fuel cell anodes. *Ind. Eng. Chem. Res.* **60**, 7826-7834 (2021).
20. Kim, M. *et al.* Understanding synergistic metal–oxide interactions of in situ exsolved metal nanoparticles on pyrochlore oxide support for enhanced water splitting. *Energy Environ. Sci.* **14**, 3053-3063 (2021).
21. Laguna-Bercero, M. A., Campana, R., Larrea, A., Kilner, J. A. & Orera, V. M. Electrolyte degradation in anode supported microtubular yttria stabilized zirconia-based solid oxide steam electrolysis cells at high voltages of operation. *J. Power Sources* **196**, 8942-8947 (2011).
22. Chen, L., Xu, J., Wang, X. & Xie, K. $Sr_2Fe_{1.5+x}Mo_{0.5}O_{6-\delta}$ cathode with exsolved Fe nanoparticles for enhanced CO_2 electrolysis. *Int. J. Hydrog. Energy.* **45**, 11901-11907 (2020).
23. Bai, L. *et al.* New insight into the doped strontium titanate cathode with in situ exsolved nickel nanoparticles for electrolysis of carbon dioxide. *Adv. Mater. Interfaces* **8**, 2001598 (2020).
24. Lv, H. *et al.* In situ investigation of reversible exsolution/dissolution of CoFe alloy nanoparticles in a Co-doped $Sr_2Fe_{1.5}Mo_{0.5}O_{6-\delta}$ Cathode for CO_2 Electrolysis. *Adv. Mater.* **32**, e1906193 (2020).
25. Lv, H. *et al.* In situ exsolved $FeNi_3$ nanoparticles on nickel doped $Sr_2Fe_{1.5}Mo_{0.5}O_{6-\delta}$ perovskite for efficient electrochemical CO_2 reduction reaction. *J. Mater. Chem. A* **7**, 11967-11975 (2019).

26. Choi, J. *et al.* Highly efficient CO_2 electrolysis to CO on Ruddlesden–Popper perovskite oxide with in situ exsolved Fe nanoparticles. *J. Mater. Chem. A* **9**, 8740-8748 (2021).
27. Tian, Y. *et al.* Boosting CO_2 electrolysis performance by calcium oxide-looping combined with in situ exsolved Ni-Fe nanoparticles based on symmetrical solid oxide electrolysis cell. *J. Mater. Chem. A* **8**, 14895-14899 (2020).
28. Xi, X., Fan, Y., Zhang, J., Luo, J.-L. & Fu, X.-Z. In situ construction of hetero-structured perovskite composites with exsolved Fe and Cu metallic nanoparticles as efficient CO_2 reduction electrocatalysts for high performance solid oxide electrolysis cells. *J. Mater. Chem. A* **10**, 2509-2518 (2022).
29. Ding, S. *et al.* A-site deficient perovskite with nano-socketed Ni-Fe alloy particles as highly active and durable catalyst for high-temperature CO_2 electrolysis. *Electrochim. Acta* **335**, 135683 (2020).
